# The predictive value of urine specific gravity in the diagnosis of vasovagal syncope in children and adolescents

**DOI:** 10.1186/s13052-021-01043-2

**Published:** 2021-04-17

**Authors:** Ping Liu, Xingfang Zeng, Wanzhen Mei, Yuwen Wang, Runmei Zou, Cheng Wang

**Affiliations:** 1 Department of Pediatric Cardiovasology, Children’s Medical Center, The Second Xiangya Hospital, Central South University, No.139 Renmin Middle Road, Changsha, 410011 Hunan China; 2 Department of Clinical Nursing, The Second Xiangya Hospital, Central South University, Changsha, 410011 Hunan China

**Keywords:** Urine specific gravity, Vasovagal syncope, Children, Adolescents

## Abstract

**Background:**

Vasovagal syncope (VVS) is a kind of common neurally mediated syncope in children and adolescents. Decreased blood volume is one of the pathogenesis of VVS. The diagnosis of VVS is mainly based on head-up tilt test (HUTT), but some complications may easily occur when HUTT induces syncope. To find a simple and safe VVS diagnosis method can improve the VVS diagnosis efficiency.

**Aims of the study:**

This was a prospective study. The study will explore the predictive value of urine specific gravity (USG) in the diagnosis of VVS in children and adolescents.

**Patients and methods:**

Ninety-seven cases (43 males and 54 females, aged 4 to 16 years old, with an average age of 10.91 ± 2.18 years old) hospitalized due to unexplained premonitory syncope or syncope and diagnosed with VVS through HUTT from September 2014 to September 2018 were selected as VVS group. During the same period, 91 cases of children and adolescents, including 45 males and 46 females, aged from 5 to 15 years old, who underwent a healthy examination were matched as a control (control group). USG was measured in both groups.

**Results:**

The USG of VVS group was significantly lower than that of the control group (*P <* 0.01), and USG of females was lower than that of males in VVS group (*P =* 0.045). The sensitivity and specificity of USG in predicting VVS were evaluated by ROC curve. The area under the ROC curve was 0.751, standard error was 0.035, and 95% CI (0.683, 0.819) suggested that USG was of moderate predictive value in the diagnosis of VVS. As cut-off value of USG was 1.0185, the sensitivity and specificity and diagnostic coincidence rate of VVS were 74.39, 66.04 and 69.68%, respectively.

**Conclusion:**

There are less USG in children and adolescents with VVS, especially lower USG in females. Therefore, USG has predictive value in the diagnosis of VVS in children and adolescents.

## Introduction

Vasovagal syncope (VVS) is a kind of common hemodynamic type of neurally mediated syncope in children and adolescents, tends to occur in the long standing, a sudden position change (eg. from the squat or sitting position suddenly to the standing position), and stuffy environment, etc. The process of syncope can lead to falling blood pressure (BP) and/or slower heart rate (HR), characterized by transient and self-limited consciousness disorders due to transient cerebral insufficiency of blood supply, accompanied by falling down to the ground because of loss of muscle tone which maintains body posture [1]. VVS can be divided into three hemodynamic types: vasoinhibitory (VVS-VI), cardioinhibitory (VVS-CI) and mixed (VVS-M) type. Its incidence is closely related to the low blood volume in patients [2], and low blood volume can cause vasovagal reflex. When the position changes suddenly, volume load of left ventricular is reduced, returned blood volume is decreased and ventricle is underfilling, causing hyperexcitability of sympathetic nerves, excess shrinking of cardiac ventricle, stimulation of the mechanical baroreceptor C fibers in the posterior inferior wall of the left ventricle, transmitting excitement to the brain stem, triggering diminished sympathetic activity and raised vagal activity, leading to abnormal bradycardia, decreased peripheral vascular resistance, fall of BP and cerebral hypoperfusion, finally resulting in syncope. Generally, VVS patients have a 24 h decrease in urinary sodium [3], and 24 h-urinary sodium is correlated with the severity of VVS. The lower the 24 h-urinary sodium is, the more severe the VVS symptoms are [4]. Therefore, measures to increase water and salt intake should be taken in treatment [5–7]. The diagnosis of VVS is mainly based on head-up tilt test (HUTT), but temporary aphasia, severe arrhythmia, convulsions, psychological fear and other complications may easily occur when HUTT induces syncope [1], and thus HUTT could only be carried out in larger hospitals. To find a simple and safe VVS diagnosis method can improve the VVS diagnosis efficiency of children and adolescents in primary hospitals.

Urine specific gravity (USG) is under the condition of 4 °C the weight ratio of the urine to the pure water with the same volume, it depends on the concentration of the urinary solute, and is proportional to the total solids. The USG of normal people may fluctuate due to differences in diet, water drinking, sweating and urination, etc. The USG of infants is usually lower than that of adults. Under pathological conditions, urine contains more proteins, glucose, ketone bodies and various cells, which could increase the USG. Clinically, the USG can be used to judge the fluid status in human body [8], predict the incidence of acute ischemic stroke [9], early evaluate the hydration status of workers in extremely low humidity environment [10], identify diabetes patients with polyuria or diabetes insipidus, etc. and also as a monitoring index for the risk of urinary calculi; for example [11], the occurrence of urinary calculi can be reduced if the USG is maintained at a low level [12]. Given that the relationship between USG and VVS in children and adolescents has been barely reported, this study aims to discuss the predictive value of monitoring USG in the diagnosis of VVS in children and adolescents.

## Patients and methods

### Study population

**Fig. 1:**
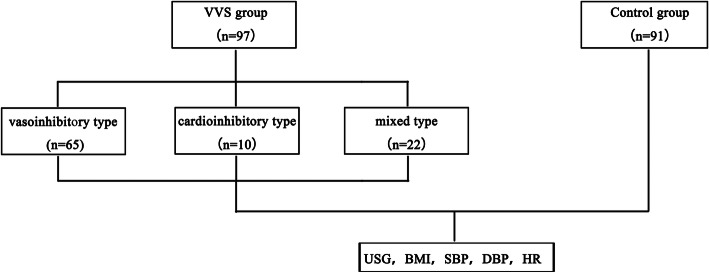
Flow chart of urine specific gravity detection

From September 2014 to September 2018, 97 cases of children and adolescents (43 males and 54 females, aged from 4 to 16 years old, with an average age of 10.9 ± 12.18 years old, including 5 cases aged from 4 to 6 years old, 70 cases aged from 7 to 12 years old, and 22 cases aged over 13 years old), hospitalized in the Department of Pediatric Cardiovasology, The Second Xiangya Hospital, Central South University, due to unexplained premonitory syncope or syncope were selected. After detailed enquiry of medical history, physical examination, blood biochemical examination (including fasting blood glucose, myocardial enzyme, etc.), routine electrocardiogram, dynamic electrocardiogram, chest X ray film, echocardiography, electroencephalogram and head magnetic resonance imaging examination, etc., excluding syncope caused by diseases of nervous system and circulatory system, immune diseases and metabolic diseases, etc., they were diagnosed with VVS through HUTT which was carried out after adequate communication with the receivers or guardians and obtaining their written informed consent (VVS group). During the same period, 91 cases of children and adolescents were randomly examined for health in the Outpatient Department of Child Health Care in our hospital were matched as the control group, 45 males and 46 females, aged from 5 to 15 years old, among which 3 were less than 7 years old, 64 were from 7 to 12 years old, and 24 were more than 13 years old. During this study, height, body mass, baseline systolic blood pressure (SBP), diastolic blood pressure (DBP) and HR were measured, body mass index (BMI, kg/m^2^) = weight (kg)/height^2^ (m^2^) was calculated. As shown in Fig. 1.

The informed consent was obtained from all the subjects directly or their guardians prior to enrollment. The study protocol was approved by the Ethics Committee of The Second Xiangya Hospital, Central South University [Issue No.12 (2014), IRB].

### Data source

#### USG test

One day after HUTT in the VVS group, 10 ml morning urine was retained, and the USG was detected by polyelectrolyte ionization method.

#### HUTT

To complete baseline head-up tilt test (BHUT) and/or sublingual nitroglycerin head-up tilt test (SNHUT).

##### The preparation before the test

Subjects should be discontinued from any cardiovascular active drugs for more than 5 half-lives prior to the test, stop taking any diet that affected autonomic function, and abstaining from eating and drinking for at least 4 h before the experiment. The test requires quiet environment, dim light and suitable temperature. The subjects and their family members (guardians) should be introduced to the precautions before the test and the possible risks during the test, and a written informed consent from the subject or his/her family (guardian) should be obtained.

##### BHUT

The examine time was arranged from 8:00 to 12:00. And the ECG and blood pressure of the right upper limb were monitored by SHUT-100 tilt test monitoring software system of Beijing Standard Medical Technology Co., Ltd. Subjects lay supine on the electric tilting bed in a quiet environment for at least 10 min with band fixed to avoid buckling of ankle joint and knee joint. Then Baseline SBP, DBP, HR and ECG were recorded. Next, the electric tilting bed was operated to place the subject at a 60 degree angle of head-up tilt position within 15 s. Changes of ECG and blood pressure were continuously monitored at the beginning of tilt and throughout HUTT process until a positive reaction occurred or the prescribed 45 min was reached. The test should be terminated and subjects should be return to the supine position as soon as a positive reaction occurred [1].

##### SNHUT

If a positive reaction never occurs within prescribed 45-min BHUT, the subject should be kept the same tilting angle on the tilting bed, simultaneously with a sublingual nitroglycerin tablet [(4–6) μg/kg (maximum≤300 μg)]. HR, BP and ECG were continuously monitored, and the test should be terminated and subjects should be return to the supine position as soon as a positive reaction occurred; If no positive reaction occurs, it should be observed until 20 min after the subject taking the drug [1].

#### Standards and types for positive reaction of VVS

Children with syncopal episodes or presyncope with any of the following responses in HUTT were considered positive patients: 1) fall of BP, 2) decrease of HR, 3) sinus arrest occurs and is replaced by junctional escape rhythm, 4) transient, secondary or more atrioventricular block and cardiac arrest for 3 s. The standard of BP decrease was SBP ≤ 80 mmHg (1 mmHg = 0.133 kPa) or DBP ≤ 50 mmHg or mean blood pressure decrease≥25%, HR decrease refers to sinus bradycardia, HR < 75 beats/min for 4–6 years old children, HR < 65 beats/min for 6–8 years old children, HR < 60 beats/min for those older than 8 years. VVS was classified into three types according to the changes in BP and HR associated with positive reactions during HUTT: 1) VVS-VI: BP drops dramatically and no significant change in HR, 2) VVS-CI: HR decreases obviously and no significant change in BP, 3) VVS-M: HR and BP both are significantly decreased [1].

### Statistical methods

The SPSS 22.0 software was used for statistical analyst, the measurement data were expressed as mean ± standard deviation $\left(\overline{x}\pm s\right)$, *t* test was used for comparison between two groups, One-Way ANOVA was used for analysis among three groups, and χ^2^ test was to compare the enumeration data between groups. The receiver operating characteristic curve (ROC) was adopted to evaluate the sensitivity and specificity of USG in predicting VVS and area under the curve (AUC) indicated the predictive ability of USG. AUC of 0.5 ~ 0.7 indicates low predictive ability, 0.7 ~ 0.9 is moderate predictive ability, and > 0.9 indicates high predictive ability. When the Youden index (the sum of sensitivity and specificity minus 1) is the maximum, the sensitivity and specificity will reach the best, and this node was selected as the cut-off value of the prediction index. *P* < 0.05 was considered statistically significant in difference.

## Results

### Comparison of general data

The difference in age, height, body mass, BMI, baseline SBP and DBP between the VVS group and the control group was nonsignificant (*P* > 0.05), baseline HR of the VVS group was higher than that of the control group (*P =* 0.001), as shown in Table 1. The difference in age, BMI, DBP, HR among the three hemodynamic types: VVS-VI, VVS-CI and VVS-M of the VVS group was nonsignificant (*P* > 0.05), height, body mass and SBP of VVS-VI group was lower than that of VVS-CI and VVS-M groups (*P =* 0.001), as shown in Table 2.

**Table 1:** Comparison of general information between the VVS group and the control group $\left(\overline{x}\pm s\right)$

Group	M/F	Age (years)	Height (cm)	Body mass (kg)	BMI (kg/m^2^)	SBP (mmHg)	DBP (mmHg)	HR (beats/min)
Control group (*n* = 91)	45/46	11.07 ± 2.02	145.71 ± 13.12	40.17 ± 12.99	18.53 ± 3.86	106.67 ± 8.24	67.03 ± 7.50	78.97 ± 7.91
VVS group (*n* = 97)	43/54	10.91 ± 2.18	148.42 ± 14.90	39.32 ± 12.19	17.54 ± 3.06	106.94 ± 8.68	67.00 ± 8.09	84.11 ± 11.00
*t*		0.517	1.301	− 0.458	−1.953	0.205	− 0.023	3.370
*P*		0.605	0.195	0.647	0.052	0.838	0.982	0.001

**Table 2:** Comparison of general information of different hemodynamic types of VVS $\left(\overline{x}\pm s\right)$

Group	n	Age (years)	Height (cm)	Body mass (kg)	BMI (kg/m^2^)	SBP (mmHg)	DBP (mmHg)	HR (beats/min)
VVS-VI group	65	10.55 ± 2.31	145.42 ± 15.10	36.37 ± 10.36	17.03 ± 2.87	105.43 ± 9.28	66.06 ± 8.14	83.98 ± 10.68
VVS-CI group	10	11.50 ± 2.55	154.90 ± 16.18	46.40 ± 18.46	18.54 ± 4.02	108.20 ± 7.89	65.80 ± 6.53	83.60 ± 9.44
VVS-M group	22	11.68 ± 1.25	154.32 ± 11.10	44.82 ± 11.12	18.56 ± 2.88	110.82 ± 5.65	70.32 ± 8.04	84.73 ± 12.91
*F*		2.706	4.257	6.494	2.760	3.449	2.468	0.049
*P*		0.072	0.017	0.002	0.068	0.036	0.090	0.953

### USG comparison

**Fig. 2:**
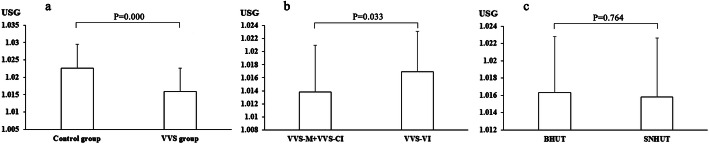
Comparison of urine specific gravity in different groups. **a** Comparison between VVS group and control group; **b** Comparison between VVS-M and VVS-CI groups and VVS-VI; **c** Comparison between BHUT and SNHUT

**Fig. 3:**
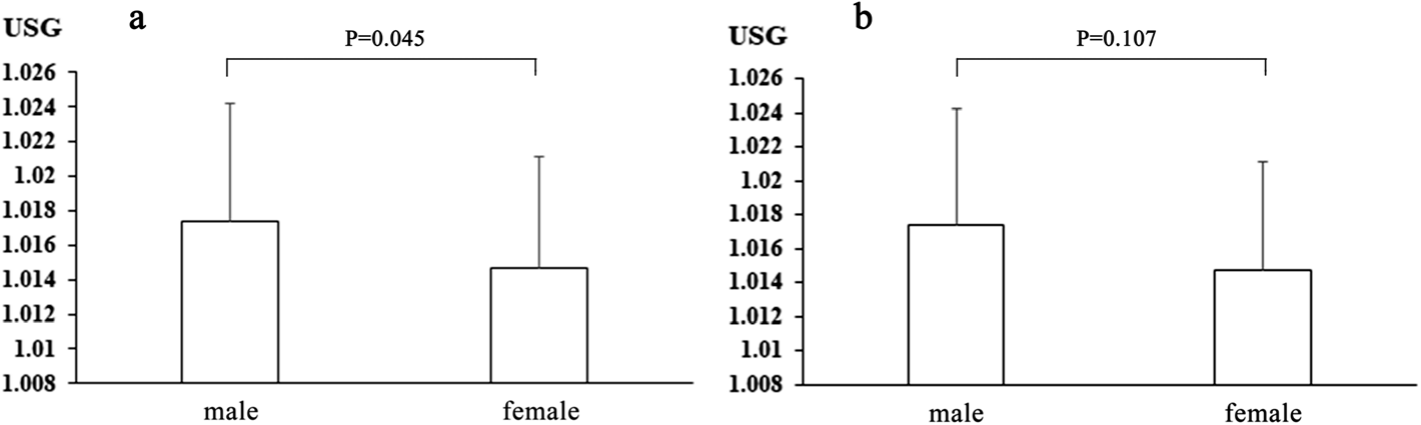
Comparison of urine specific gravity of different genders. **a** VVS group; **b** control group

USG was lower in the VVS group than in the control group (*P =* 0.000), USG of VVS-M and VVS-CI groups was lower than that of VVS-VI group (*P =* 0.033), the difference in BHUT group and SNHUT group was nonsignificant (*P =* 0.764), as shown in Fig. 2. In the VVS group, USG of females was lower than that of males (*P =* 0.045), the difference in USG between males and females in the control group was nonsignificant (*P =* 0.107), as shown in Fig. 3.

### ROC curve

**Fig. 4:**
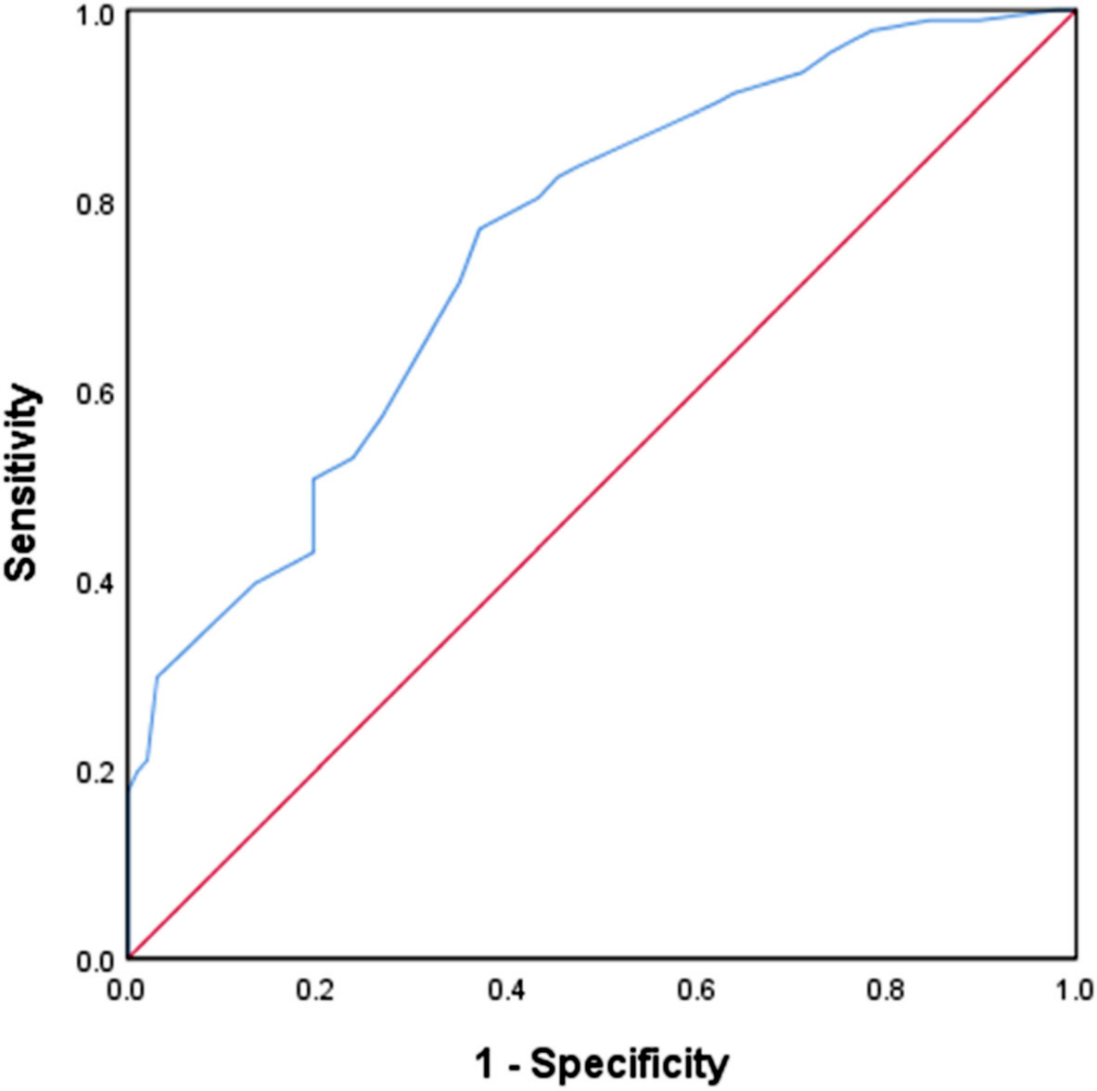
ROC Curve on the Predictive Value of USG in the VVS Diagnosis in Children and Adolescents. Notes: ROC curve on the predictive value of VVS diagnosis in children and adolescents with USG as the cut-off value. The vertical axis denotes the sensitivity of USG to the predictive value of VVS diagnosis, and the horizontal axis indicates the false positive rate (1-specificity), the solid line of 45°coordinate in the figure is criterion-referenced line, indicating that the sensitivity and the false positive rate is equal, no predictive value. The farther the curve is to the upper left of the reference line, the higher the predictive value is. The AUC represents the predictive value of USG in the diagnosis of VVS. The AUC value of 0.5 ~ 0.7 is low, 0.7 ~ 0.9 is medium, and > 0.9 is high

The receiver operating characteristic curve (ROC) was used to evaluate the sensitivity and specificity of USG in predicting VVS. The area under the ROC curve was 0.751, standard error was 0.035, and 95% confidence interval (0.683, 0.819) suggested that USG had a moderate predictive value for the diagnosis of VVS. When cut-off value of USG was 1.0185, the sensitivity and specificity and diagnostic coincidence rate of predicting VVS were 74.39, 66.04 and 69.68%, respectively. as shown in Fig. 4.

## Discussion

USG and urine color are widely used in clinical practice [13]. USG can be used as a marker to monitor the level of moderate drinking water in healthy people and monitor the internal fluid status of athletes. Wilcoxson et al. [8] reported that dynamic monitoring of USG had a good effect on fluid retention and hydration in male runners. Osterberg et al. [14] covered that USG was used to determine the pregame fluid intake of American Basketball Association (NBA) athletes, when pregame USG was ≤1.020, athletes should be ensured to supply adequate water. And Stover et al. [15] found that the USG of exercisers could determine water dehydration in the body and did not change with time and place before physical exercise. If the USG of exercisers was≥1.020, it suggested that 46% of exercisers might be dehydrated. Liu et al. [16] reported that USG could assist in differentiating SARS-CoV-2 patients from healthy people, and the USG of the former was lower.

Patients with VVS may be at low blood volume status. El-sayed et al. [17] conducted a double-blind randomized controlled study with oral rehydration salts (ORS) treatment on 20 VVS adult patients and found that, after more than 2 months of follow-ups, the clinical symptoms of VVS patients were significantly improved, especially in patients with previous salt intake< 170 mmoL/d, their blood volume increased and so did their tolerance to erectness, with more apparent symptoms improvement. Younoszai et al. [18] gave oral isotonic saline intervention to 58 VVS children, and 90% (52/58) of the children had complete relief of symptoms, suggesting that oral liquid therapy was effective in treating VVS children. Li et al. [6] reported that oral rehydration salt (ORS) was mainly effective for children with VVS-VI. Wen et al. [19] reported that ORS was an effective measure to treat VVS in children and adolescents, and recommended that ORS treatment course be 2 months.

Some biomarkers may have predictive value for the diagnosis and intervention effect of VVS in children. Zhang et al. [20] reported changes in creatine kinase (CK) and creatine kinase-MB (CK-MB) of VVS group (*n* = 218) and control group (*n* = 129) in children and adolescents, and found that the decrease of CK and CK-MB had a protective effect on VVS, when CK was 89.65 U/L, the sensitivity and specificity of diagnosing VVS were 50.90 and 78.30%, respectively. When CK-MB was 23.94 U/L, the sensitivity and specificity of diagnosing VVS were 71.10 and 66.70%, respectively. Tao et al. [21] reported therapeutic response of baroreflex sensitivity (BRS) in predicting VVS patients to metoprolol, and compared with non-responders, patients with reaction showed a significant increase in BRS value in supine position. When BRS cut-off value was 10 ms/mmHg, the sensitivity and specificity of predicting an intervention response were 82 and 83%. Tao et al. [22] also reported the predictive value of acceleration index (AI) in postural training effect of VVS children, the sensitivity and specificity of predicting effective training were 85.0 and 69.2% when AI cut-off value was 26.77. Tao et al. [23] also observed that BMI predicted the intervention effect of VVS children on ORS, when BMI cut-off value was 18.9 kg/m^2^, the sensitivity and specificity for predicting the effective intervention of ORS in VVS was 83 and 73%. In this study, the USG of children and adolescents with VVS was significantly lower than that of healthy control children (*P* < 0.01), the area under the ROC curve was 0.751, standard error was 0.035, and 95% confidence interval (0.683, 0.819) suggested that USG was of moderate predictive value in the diagnosis of VVS. When cut-off value of USG was 1.0185, the sensitivity and specificity and diagnostic coincidence rate of predicting VVS were 74.39, 66.04 and 69.68%, respectively. This result is similar to the effect of CK and CK-MB in predicting and diagnosing VVS reported by Zhang et al. [20]. This study further showed that the USG of females in the VVS group was lower than that of males (*P* < 0.05), which may be related to factors such as females’ low-salt diets and males’ relatively high fluid loss due to excessive activity [24].

However, USG test is more convenient and more suitable for clinical operation. The results of this study suggested that the USG index could better reflect the amount of water in VVS children and adolescents, and the USG decrease of VVS children is consistent with the previous findings on the pathogenesis of insufficient capacity load in the human body, which guided to increase the patients’ intake of water and salt in clinical practice, and the effect of water and salt supplementation could be monitored by USG [25].

## Conclusion

USG has a certain predictive value in the diagnosis of VVS in children and adolescents and the USG test is simple and suitable for all levels of medical institutes to predict the diagnosis of VVS in children and adolescents.

## Data Availability

The datasets used and/or analyzed during the current study are available from the corresponding author on reasonable request.

## Abbreviations

AI: Acceleration index
AUC: Area under the curve
BP: Blood pressure
BMI: Body mass index
BHUT: Basic head-up tilt test
BRS: Baroreflex sensitivity
CK: Creatine kinase
CK-MB: Creatine kinase-MB
DBP: Diastolic blood pressure
ECG: Electrocardiogram
HR: Heart rate
HUTT: Head-up tilt test
IRB: Institutional review board
ORS: Oral rehydration salt
ROC: Receiver operating characteristic curve
SBP: Systolic blood pressure
SNHUT: Sublingual nitroglycerin head-up tilt test
USG: Urine specific gravity
VVS: Vasovagal syncope
VVS-VI: Vasovagal syncope vasoinhibitory type
VVS-CI: Vasovagal syncope cardioinhibitory type
VVS-M: Vasovagal syncope mixed type
